# Cluster randomised trials in the medical literature: two bibliometric surveys

**DOI:** 10.1186/1471-2288-4-21

**Published:** 2004-08-13

**Authors:** J Martin Bland

**Affiliations:** 1 Department of Health Sciences, University of York, York YO10 5DD, United Kingdom

## Abstract

**Background:**

Several reviews of published cluster randomised trials have reported that about half did not take clustering into account in the analysis, which was thus incorrect and potentially misleading. In this paper I ask whether cluster randomised trials are increasing in both number and quality of reporting.

**Methods:**

Computer search for papers on cluster randomised trials since 1980, hand search of trial reports published in selected volumes of the *British Medical Journal *over 20 years.

**Results:**

There has been a large increase in the numbers of methodological papers and of trial reports using the term 'cluster random' in recent years, with about equal numbers of each type of paper. The *British Medical Journal *contained more such reports than any other journal. In this journal there was a corresponding increase over time in the number of trials where subjects were randomised in clusters. In 2003 all reports showed awareness of the need to allow for clustering in the analysis. In 1993 and before clustering was ignored in most such trials.

**Conclusion:**

Cluster trials are becoming more frequent and reporting is of higher quality. Perhaps statistician pressure works.

## Background

Cluster randomised trials are those where research subjects are not allocated to treatments independently, but as a group. For example, in a study of counselling patients on physical activity in general practice, practices were allocated to counselling or control and patients aged 40–79 years who attended during a five day period and who did not take regular exercise were invited to take part. Patients in the same practice received the same treatment, counselling or usual care, depending on how the practice was allocated. [1] The group of patients within the general practice formed a cluster.

Members of a cluster will be more like one another than they are like members of other clusters and we need to take this into account in the analysis, and preferably the design, of the study. Methods which ignore clustering may mislead, because they assume that all subjects provide independent observations. Applying simple statistical methods to such data, without taking the clustering into account, can lead to confidence intervals which are too narrow and P values which are too small.

There has been an increasing interest in cluster randomised trials over the past 20 years. For example, by the end of 2003 the *British Medical Journal *Statistics Notes on this topic [2-7] had been cited 121 times.

There have been several reviews of published cluster randomised trials [8-13] (Table 1). All but Puffer *et al. *[10] reported that very few trials had sample size calculations which included clustering and about half took clustering into account in the analysis, fewer in the African studies reported by Isaakidis and Ioannidis. [11] Puffer *et al. *[10] did not mention whether trials failed to take clustering into account in the analysis. My own review of their trials as listed on the *British Medical Journal *website found that only 3 out of 36 ignored clustering. The review of the *American Journal of Public Health *and *Preventive Medicine *in 1998 – 2002 [13] is especially interesting because it attempted to replicate an earlier study [9] in the same journals. There was an increase in the number of reports of cluster randomised trials: 12.3 studies were reported per year in 1998 – 2002 compared to 5.3 studies per year in 1990 – 1993. [9] The quality of the analysis may have improved, but such assessments are subjective and very difficult to compare between reviews.

**Table 1:** Some reviews of published cluster randomised trials

Authors	Source of trials	Years	Clustering allowed for in sample size	Clustering allowed for in analysis
Donner *et al. *[8]	16 non-therapeutic intervention trials	1979 – 1989	<20%	<50%
Simpson *et al. *[9]	21 trials from *American Journal of Public Health *and *Preventive Medicine*	1990 – 1993	19%	57%
Isaakidis and Ioannidis [11]	51 trials in Sub-Saharan Africa	1973 – 2001 (half post 1995)	20%	37%
Puffer *et al. *[10]	36 trials in *British Medical Journal*, *Lancet*, and *New England Journal of Medicine*	1997 – 2002	56%	92% ^a^
Eldridge *et al. *[12]	152 trials in primary health care	1997 – 2000	9%	59%
Varnell *et al. *[13]	60 trials in *American Journal of Public Health *and *Preventive Medicine*	1998 – 2002	20%	54% (all analyses) + 25% (some analyses only)

^a ^My review of trials identified by Puffer *et al. *[10]

It is understandable that papers do not report sample size calculations, as often these are omitted from papers entirely, sometimes by the request of the journal to save space. It can be argued (though I would not do so) that once we have carried out a study, the sample size calculations are not particularly informative. Analysis which ignores the clustering, however, can be highly misleading, finding significant differences where there are none. We may have incorrect conclusions in the literature, which are then uncritically repeated and become false knowledge.

We should not be surprised that clustering is ignored. In the past, few textbooks have cautioned against this and the assumption of independence of observations is seldom stressed. Many statisticians will admit to having incorrectly ignored clustering in the analysis of clustered designs, including myself when I was younger and more ignorant than today. However, it can be very important.

In this paper I attempt to chart the changes in both the number of cluster randomised trials reported and the proportion of these reports where clustering has been taken into account in the analysis.

## Methods

I first carried out a search on the ISI Web of Science, looking for papers on cluster randomisation and reports of trials. I classified these by type (trial report or methodological article), year of publication, and journal.

To identify cluster randomised trials we have to read the papers. We cannot tell whether a trial is cluster randomised from title, keywords, or abstract. Many authors are not aware of the importance of clustering and do not mention it. In this paper I report the results of a hand search of the *British Medical Journal*. I identified and scanned all papers reporting trials for the years 1983, 1988, 1993, 1998, and 2003, recording any where subjects were allocated in clusters. I excluded any studies where subjects were not allocated to groups by the investigator, for example several comparisons of fund-holding and non-fund-holding general practices.

For each trial identified, I noted whether clustering had been taken into account in the analysis. There are several approaches which can be used to allow for clustering. The easiest is to calculate a summary statistic for each cluster. [4] This is usually a mean for a continuous outcome or a proportion for a dichotomous outcome. We can also use robust variance estimates, general estimating equation models (GEEs), multilevel modelling, Bayesian hierarchical models, and several other techniques. Any method which takes into account the clustering should be an improvement compared to methods which do not.

I also noted whether ignoring the possible effects of clustering might have an important effect on the conclusions. Clustering may result in P values and confidence intervals which are sufficiently biased to have a major effect if any of the following are true: the cluster size is large, the number of clusters is small, or the intra-cluster correlation coefficient is large. Whether any of these applies in a trial which ignores clustering is a matter of judgement.

## Results

### A computer search for cluster randomised trials

**Figure 1:**
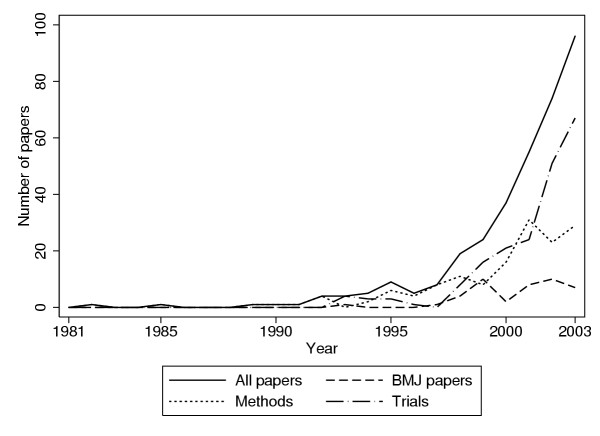
**Results of a Web of Science search. **Results of a Web of Science search on: randomi* in clusters OR cluster randomi*, up to the end of 2003.

Figure 1 shows the result of a search on the ISI Web of Science, looking for papers on cluster randomisation and reports of trials. I found that other terms, such as 'group randomised' did not work, as I got hundreds of abstracts with 'patients were in two groups, randomised to active or control treatments'.

There are many potential biases. In the first part of the period, the Web of Science database did not include abstracts, so there was less opportunity to pick up the search terms. More recently, several journals began to include a description of the trial design in the title of the paper, for example 'Effect on hip fractures of increased use of hip protectors in nursing homes: cluster randomised controlled trial'. [14] This will increase the detection rate. These design descriptions are not always correct, nor does the cluster randomised nature of the trial necessarily appear in the description. Also, many authors will not be aware of the importance of clustering and will not mention it. These factors will reduce detection. Hence this is not a thorough search and will have missed many studies, but it might give an idea of the increase in activity.

I divided the papers into those which were methodological, either educating researchers into the appropriate design and analysis of cluster randomised trials or developing new methods of analysing such trials, and those reporting actual trials. The data for 2000 and 2001 includes special issues of *Statistics in Medicine *and *Statistical Methods in Medical Research *on cluster randomisation, so there were a larger number of methodological papers than might be expected in those years. The numbers of papers found in the two categories were similar in each year before 2001: as many papers were about how to do such trials as were reports of actual trials. It is hard to believe that there are so few such trials being reported and it is likely that many have been reported without any acknowledgement of the importance of clustering.

All the papers up to 1990 are due to Donner and his colleagues. [8,15,16] However, it was impossible to identify papers which used older terminology. A paper by Cornfield [17] 'Randomisation by group: a formal analysis' includes the following statement 'Randomization by cluster accompanied by an analysis appropriate to randomization by individual is an exercise in self-deception, however, and should be discouraged.' This would not be found by the search. The book on cluster randomization by Murray [18] is called *The Design and Analysis of Group-Randomized Trials*.

The *British Medical Journal *was the journal most frequently represented in the survey, no fewer than 43 of the 332 publications found, 13%, appearing there. The next was *Statistics in Medicine *with 39 publications (12%), all methodological, then the *British Journal of General Practice *with 17 (5%), *Controlled Clinical Trials *with 16 (5%), and *Family Practice *with 10 (3%). Figure 1 shows that papers in the *British Medical Journal *reflect the literature as a whole. The first paper in the *BMJ *was a report of a trial, [19] which was followed four years later by a series of short educational articles. [3,4,6,7]

When I first did the search reported in Figure 1, I was surprised by how few trials were reported. My subjective impression was that there were many more cluster randomised trials than I had found. I therefore decided to carry out a small survey of journals to find out whether the dramatic increase shown in Figure 1 was real. As the *British Medical Journal *had most reports and had been published for many years, this was the obvious journal with which to begin.

### A survey of papers in the British Medical Journal

The results of the search are shown in Table 2. As Table 2 shows only reports of trials, it does not include all the *BMJ *papers in Figure 1, which also includes methodological papers. A list of all papers reviewed is given in the additional file: papers in the survey. Only one of the trials in survey [1] cited any of the *BMJ *Statistics Notes on clustering. [2-7]

**Table 2:** Result of a hand search for cluster randomised trials in the *British Medical Journal*

Year	Vol	Trials	Clustering ignored	Ignoring clustering judged as important	Found in Web of Science search
2003	326-7	9	0	0	5
1998	316-7	4	1(?)	1	0
1993	306-7	4	3	2	0
1988	296-7	0	0	0	0
1983	286-7	1	1	1	0

? doubtful whether clustering taken into account

The noted query relates to a paper in which the authors stated that 'Univariate comparisons were calculated by t test and χ^2 ^analysis. The role of potential covariates was explored using linear regression specified as a two level model (practice and individual) using the software package MLn'. [20] I could find no multilevel modelling in this paper, but a lot of t and χ^2 ^tests. This was a trial of community based management in failure to thrive by babies. Thirty eight primary care teams were randomly allocated to intervention or control and all children identified in the practice were offered the same intervention, so clearly clustering should be taken into account.

The trials which I regarded as failing to take the clustering into account were as follows. Russell *et al. *[21] investigated the effect of nicotine chewing gum as an adjunct to general-practitioners advice against smoking. Subjects were 'assigned by week of attendance (in a balanced design) to one of three groups (a) non-intervention controls (b) advice and booklet (c) advice and booklet plus the offer of nicotine gum.' There were 6 practices, with recruitment over 3 weeks, one week to each regime. The study was analysed by chi-squared tests. As the clusters were large, with 1938 subjects in 18 clusters, clustering should have been taken into account.

Rink *et al. *[22] investigated the impact of introducing near patient testing for standard investigations in general practice. Twelve practices were used, and some given the equipment and some not in a cross-over design. Analysis used paired t tests, unpaired t tests, odds ratios, ratios of proportions with confidence intervals, and chi squared tests, none of which took clustering into account.

In a trial of clinical guidelines to improve general-practice management and referral of infertile couples, Emslie *et al. *[23] randomised 82 general practices in Grampian region and studied 100 couples in each group. However, the main outcome measure was whether the general practitioner had taken a full sexual history and examined and investigated both partners appropriately. The cluster size may be small but the cluster effect may be large. The GP should be the unit of analysis here as opposed to the couple, as done in the paper.

The trial where I judged ignoring clustering to be unimportant had many very small clusters. Wetsteyn and Degeus [24] compared 3 regimens for malaria prophylaxis in travellers to Africa. Members of one family were allocated to one regimen and the results analysed using a chi-squared test.

Only five of the 18 trials had been found in the Web of Science search, showing that that was indeed an underestimate. However, the growth in numbers of trials is indicated by both electronic and hand searches.

## Discussion

A bibliometric survey has suggested a rapid increase in the number of cluster randomised trials, many of which appeared in the *British Medical Journal*. A hand search of the *British Medical Journal *has confirmed this increase, at least in this journal. Although the effects of clustering have often been ignored in trials, producing potentially misleading conclusions, the situation has certainly improved in the *British Medical Journal*. This has followed many articles on the topic in the Journal. Perhaps statistician pressure works.

Identification of cluster allocation is subjective. I included one year, 1998, also searched by Puffer *et al. *[10] and identified four trials where Puffer *et al. *[10] identified only one. My assessments of whether clustering has been taken into account and whether ignoring it might be important are also subjective. Nevertheless, I think that the general conclusion of increasing activity and better reporting of trials, at least in the *British Medical Journal*, is valid. Whether we would find a similar improvement in other journals is less certain. It is likely that reporting of cluster randomised trials in the *British Medical Journal *is especially good, as the journal reports many such trials, has carried many articles on their correct analysis and reporting, has a fairly rigorous statistical refereeing system, [25-27] and is generally of a relatively high methodological standard. The *BMJ*'s current statistical checklist [28] does not mention clusters, however. It would be possible to extend the survey to other journals where such trials are frequently reported, but these, too, might be more likely to adhere to sound principles of analysis and reporting than would journals where few such studies appear. The thought of hand-searching journals where no trials might be found does not appeal.

There are still many other aspects of trial reporting where improvement is possible [10,12] but the picture drawn by this survey is encouraging. Methodologists need to keep up the pressure and to extend it to specialist journals. The recently published extension of the CONSORT statement to cluster randomised trials is to be welcomed. [29] We should also pursue other types of study where the unit of analysis is doubtful, such as those involving observations of multiple body parts in the same patient or multiple measurements on the same tissue treated as independent.

## Conclusions

Cluster trials have become much more frequent since the mid 1990s. Reporting of these trials has improved and in the journal which publishes more than any other the quality had improved greatly. This improvement has followed a large number of articles advocating methods of analysis which take clustering into account, Perhaps statistician pressure works.

## Competing interests

The author has been published frequently in the *British Medical Journal*, including articles written for payment.

## Authors' contributions

J. M. Bland is the sole contributor.

## Pre-publication history

The pre-publication history for this paper can be accessed here:



## Supplementary Material

Additional File 1 **Papers in the survey **List of papers in the survey of the BMJ, Word file. Click here for file
