# Supplementary material for: Cluster randomised trials in the medical literature: two bibliometric surveys
Source: BMC Med Res Methodol. 2004 Aug 13;4:21. doi: 10.1186/1471-2288-4-21 (PMC515302; doi:10.1186/1471-2288-4-21)
Supplement: Additional File 1 — Papers in the survey List of papers in the survey of the BMJ, Word file. [file 1471-2288-4-21-S1.doc]

**Papers in the survey**

**Christian P, Khatry SK, Katz J, Pradhan EK, LeClerq SC, Shrestha SR, Adhikari RK, Sommer A,West KP.** **Effects of alternative maternal micronutrient supplements on low birth weight in rural Nepal: double blind randomised community trial.** *Brit Med J* 2003, **326**:571-574.

Coulthard MG, Vernon SJ, Lambert HJ, Matthews JNS. **A nurse led education and direct access service for the management of urinary tract infections in children: prospective controlled trial.** *Brit Med J* 2003, **327**:656-659.

**Elley CR**, **Kerse** **N**, **Arroll** **B**, **Robinson** **E**. **Effectiveness of counselling patients on physical activity in general practice: cluster randomised controlled trial.** *Brit Med J* 2003, **326**:793-796.

Emslie C, Grimshaw J, Templeton A. **Do clinical guidelines improve general-practice management and referral of infertile couples?** *Brit Med J* 1993, **306**:1728-1731.

English DR, Burton RC, del Mar CB, Donovan RJ, Ireland PD, Emery G. **Evaluation of aid to diagnosis of pigmented skin lesions in general practice: controlled trial randomised by practice.** *Brit Med J* 2003, **327**:375-378.

Glasgow NJ, Ponsonby A-L, Yates R, Beilby J, Dugdale P. **Proactive asthma care in childhood: general practice based randomised controlled trial**. *Brit Med J* 2003, **327**:659-663.

Kinmonth AL, Woodcock A, Griffin S, Spiegal N, Campbell MJ. **Randomised controlled trial of patient centred care of diabetes in general practice: impact on current wellbeing and future disease risk.** *Brit Med J* 1998, **317**:1202-1208.

**Meyer G**, **Warnke A**, **Bender** **R**, **Mühlhauser I**. **Effect on hip fractures of increased use of hip protectors in nursing homes: cluster randomised controlled trial.** *Brit Med J* 2003, **326**:76-78.

Modell M, Wonke B, Anionwu E, Khan M, Tai SS, Lloyd M, Modell B. **A multidisciplinary approach for improving services in primary care: randomised controlled trial of screening for haemoglobin disorders.** *Brit Med J* 1998, **317**:788-791.

Moore H, Summerbell CD, DC Greenwood, Tovey P, Griffiths J, Henderson M, Hesketh K, Woolgar S, Adamson AJ. **Improving management of obesity in primary care: cluster randomised trial.** *Brit Med J* (2003, **327**:1085 -

Nutbeam D, Macaskill P, Smith C, Simpson JM, Catford J. **Evaluation of 2 school smoking education-programs under normal classroom conditions.** *Brit Med J* 1993, **306**: 102-107.

Rink E, Hilton S, Szczepura A, Fletcher J, Sibbald B, Davies C, Freeling P, Stilwell J. **Impact of introducing near patient testing for standard investigations in general-practice.** *Brit Med J* 1993, **307**:775-778.

Russell MAH, Merriman R, Stapleton J, Taylor W. **Effect of nicotine chewing gum as an adjunct to general-practitioners advice against smoking.** *Brit Med J* 1983, **287:**1782-1785.

Smeeth L, Fletcher AE, Hanciles S, Evans J, Wormald R. **Screening older people for impaired vision in primary care: cluster randomised trial.** *Brit Med J* 2003, **327**:1027-1030.

Toroyan T, Roberts I, Oakley A, Laing G, Mugford M, Frost C. **Effectiveness of out-of-home day care for disadvantaged families: randomised controlled trial.** *Brit Med J* 2003, **327**:906-909.

Wetsteyn JCFM, Degeus A. **Comparison of 3 regimens for malaria prophylaxis in travelers to East, Central, and Southern Africa.** *Brit Med J* 1993, **307**:1041-1043.

Wright CM, Callum J, Birks E, Jarvis S. **Effect of community based management in failure to thrive: randomised controlled trial.** *Brit Med J* 1998, **317**:571-574.

Wyatt JC, Paterson-Brown S, Johanson R, Altman DG, Bradburn MJ, Fisk NM. **Randomised trial of educational visits to enhance use of systematic reviews in 25 obstetric units.** *Brit Med J* 1998, **317**:1041-1046.
